# Mindful Nonreactivity, Anxiety, Depression, and Perceived Stress as Mediators of the Mindfulness Virtual Community Intervention—Pathways to Enhance Mental Health in University Students: Secondary Evaluation of Two Randomized Controlled Trials With Student Participants

**DOI:** 10.2196/65853

**Published:** 2025-08-18

**Authors:** Meysam Pirbaglou, Christo El Morr, Farah Ahmad, Paul Ritvo

**Affiliations:** 1 School of Kinesiology and Health Science, Faculty of Health, York University, Toronto, ON, Canada; 2 School of Health Policy & Management, Faculty of Health, York University, Toronto, ON, Canada; 3 School of Kinesiology and Health Science, Department of Psychology, Faculty of Health, York University, 4700 Keele Street, Toronto, ON, M3J 1P3, Canada

**Keywords:** online interventions, student mental health, randomized controlled trial, mindfulness cognitive behavioral therapy, mediation analysis, mental health, questionnaire, anxiety, depression, stress, quality of life, virtual community, undergraduate, college, CBT, health intervention, young people, cognitive behavioral therapy, online therapy

## Abstract

**Background:**

Mindfulness-based interventions (MBIs) are widely used in mental health promotion and treatment. Despite widespread evidence of effectiveness with different populations and delivery modes, there are sparse findings concerning the mechanisms of action in MBIs.

**Objective:**

The objective of this paper was to understand the mediators of the Mindfulness Virtual Community (MVC) intervention, an 8-week, multicomponent, online mindfulness and cognitive-behavioral therapy (M-CBT) intervention, based on a secondary evaluation of 2 randomized controlled trials (RCTs) with student participants.

**Methods:**

Mediation analysis, using structural equation modeling, was used to assess direct and indirect relationships between study group (ie, intervention or wait list control) and outcomes. Consistent with the intervention’s theoretical perspective and direct effects paths, a model was specified to evaluate whether mindful nonreactivity, as evaluated by the 5-factor mindfulness questionnaire, mediated the effect of MVC intervention on anxiety and depression (as symptom-driven outcomes), and perceived stress and quality of life (as functional outcomes). The model included additional mediating paths for perceived stress through anxiety and depression, and for quality of life through anxiety, depression, and perceived stress. The model was thereafter extended, adjusting for pre-intervention differences in mindfulness (ie, observing, describing, activity with awareness, nonjudgment, and nonreactivity) facets.

**Results:**

Direct (nonmediated) effects indicated statistically significant differences at 8 weeks between the MVC and waitlist control (WLC) groups on depression (−1.72; *P*=.002), anxiety (−3.40; *P*=.001), perceived stress (−2.44; *P*<.001), quality of life (4.31; *P*=.005), and the nonreactivity facet of mindfulness (1.63; *P*<.001), in favor of the MVC intervention. Mediation analysis supported the mediating role of the nonreactivity facet of mindfulness, depression, anxiety, and perceived stress through single and sequential mediation paths. Results indicated good fit characteristics for the main (comparative fit index [CFI]=.99; root-mean-square error of approximation [RMSEA]=.05; standardized root-mean-square residual [SRMR]=.05) and extended (CFI=.99; RMSEA=.04; SRMR=.04) models.

**Conclusions:**

This research underscores the importance of mindful nonreactivity, depression, and anxiety as key mediators of MVC intervention benefits.

## Introduction

Mindfulness-based interventions (MBIs) integrate mindfulness principles and mindfulness meditation practice in established psychotherapies (eg, cognitive behavioral therapies), and other health interventions (eg, stress reduction, mindful eating, and mindful physical activity) [1-9]. Despite differences in theoretical perspective and the extent of formal mindfulness meditation practice, MBIs emphasize development of (1) attention regulation, including moment-to-moment awareness of internal and external experiences and (2) an open, nonjudgmental orientation toward these experiences [10-12]. MBIs have demonstrated effectiveness across multiple psychological outcomes (eg, anxiety, depression, pain, etc), populations (eg, children, youth, and adults), and delivery modes (eg, face-to-face and online), with Cohen *d* effect sizes ranging between 0.10 and 0.89 [1].

The search for mechanisms of action (ie, active ingredients), in addition to evaluations of clinical effectiveness, is essential for a thorough assessment of MBIs. Mediation analyses, which are widely applied in evaluations of physical and mental health interventions [13-15], offer a useful framework for examining patterns of direct and indirect (ie, mediated) interrelations among predictors and outcomes. Accordingly, mediation analyses play a critical role in the development of empirically driven theories of how interventions deliver benefits.

Previous efforts in identifying mediators of MBIs indicate that various psychological constructs account for beneficial mental health impacts. Specific mediators, across MBIs following different theoretical perspectives, include: mindfulness [16-26], self-compassion [16,18,20,23,27], decentering [19,24,28], psychological flexibility [25,29], and acceptance [24,29,30], in addition to reduced rumination [21,26,31], worry [17,21,31], experiential avoidance [28,32], cognitive defusion [33], anxiety sensitivity [33], positive or negative affect [17], dysfunctional attitudes [28], and cognitive-emotional reactivity [16,27,34,35].

Mindfulness, as a key mechanism of action in MBIs, is regarded in current research as both a disposition [36,37], and a capacity to regulate attention and emotion in relating to experiences with an open, accepting, and nonavoidant attitude [11,38]. This capacity, developed in mindfulness meditation and mindfulness skills training, has been differently operationalized in current literature [39,40]. For example, the Five Facet Mindfulness Questionnaire (FFMQ) [40], a widely used measure of mindfulness, operationalizes mindfulness as a multidimensional construct, consisting of five conceptually distinct but inter-related facets. These include: the observing (OBS), describing (DES), acting with awareness (AAW), nonjudgment (NJU), and nonreactivity (NRE) facets. These facets show different patterns of association with indices of mood and sensitivities to change in response to treatments [41-43]. However, relatively few studies have examined mindfulness facets, as conceptualized in the FFMQ, as mediators of MBIs [21-23,44]. Further examination of FFMQ-SF facets with respect to differences in sensitivity to change, norms in different populations, and mediating influence is warranted.

Using data from the Mindfulness Virtual Community (MVC) intervention, this study aims to evaluate the role of FFMQ facets as mediators of intervention effects on depression, anxiety, perceived stress, and quality of life outcomes. The MVC intervention was an 8-week web-based intervention based on mindfulness and cognitive-behavior therapy (M-CBT) principles that addressed psychological distress in university students [45,46]. This intervention comprised (1) a total of 16 psycho-educational and mindfulness meditation instruction videos (with associated printed text), (2) an anonymous peer discussion forum, and (3) an anonymous counselor-moderated video discussion session. Results from previous randomized controlled trials (RCTs) of the MVC intervention (2017‐2019) have been published [47-49], demonstrating effectiveness at improving variables reflecting depression [47,48], anxiety [47,48], mindfulness [47,48], quality of life [47], and perceived stress [47,49], when compared to waitlist control (WLC) groups.

## Methods

### Participants

Participants were undergraduate students from a large university in Toronto, Ontario, Canada, who participated in the MVC intervention during the 2017 and 2018 academic years. Details on study design, recruitment, procedures, and psychometric questionnaires have been previously published [47-49]. Given identical recruitment and program administration in the 2 study cohorts (2017‐2018), data from the 2017 and 2018 cohorts were aggregated for this analysis. Data from a third RCT, which was undertaken during a university-wide labor strike in 2019, were not included in this analysis.

### Measures

Participants were administered several questionnaires, at baseline and at 8-week post intervention: (1) The Five Facet Mindfulness Questionnaire-Short Form (FFMQ-SF), a 24-item, 5-point Likert scale (item range: 1‐5, total score: 24‐120), which evaluated 5 mindfulness facets [50]. These included the OBS (4 items), DES (5 items), AAW (5 items), NJU (5 items), and NRE (5 items) facets. Depression was assessed by the Patient Health Questionnaire-9 (PHQ-9) [51], a 4-point Likert scale (item range: 0‐3, total score: 0‐21), with higher scores indicating higher levels of depressive symptoms. Anxiety was evaluated by the Beck Anxiety Inventory (BAI) [52], a 21-item 4-point Likert scale (item range: 0‐3, total score: 0‐63), with higher scores indicating higher levels of anxiety symptoms. Perceived stress was evaluated using the Perceived Stress Scale (PSS), a 10-item, 4-point Likert scale (item range: 0‐4, total score: 0‐40) [53]. Quality of life was evaluated by the 16-item, 7-point (item range: 1‐7, total score: 16‐112) Quality of Life Scale (QOLS) [54].

### Statistical Analysis

Following calculations of descriptive statistics for demographic and psychological characteristics, possible between-group differences at preintervention were evaluated using independent samples *t* test for numerical variables, and chi-square tests of independence for categorical variables. Missing data were minimal (ie, <10‐15% of total sample), and there were no statistically significant differences in study outcomes at preintervention between participants who completed the study and those who dropped out. To estimate missing observations, we used multiple imputations and produced 10 imputed datasets. Statistical analyses were performed using SPSS (version 26.0; IBM Corp) [55]. Multiple imputation was performed in RStudio (version 4.2; Posit) [56], using the Multivariate Imputation by Chained Equations (MICE) [57] package, and mediation analysis, using a structural equation modeling approach, was performed by lavaan [58], and lavaan.mi [59] packages. Lavaan.mi provides pooled parameter estimates and CIs based on multiple imputed datasets, according to Rubin rules. Monte Carlo Simulations based on 20,000 iterations were used to determine the 95% CIs.

### Model Specification

**Figure 1.**
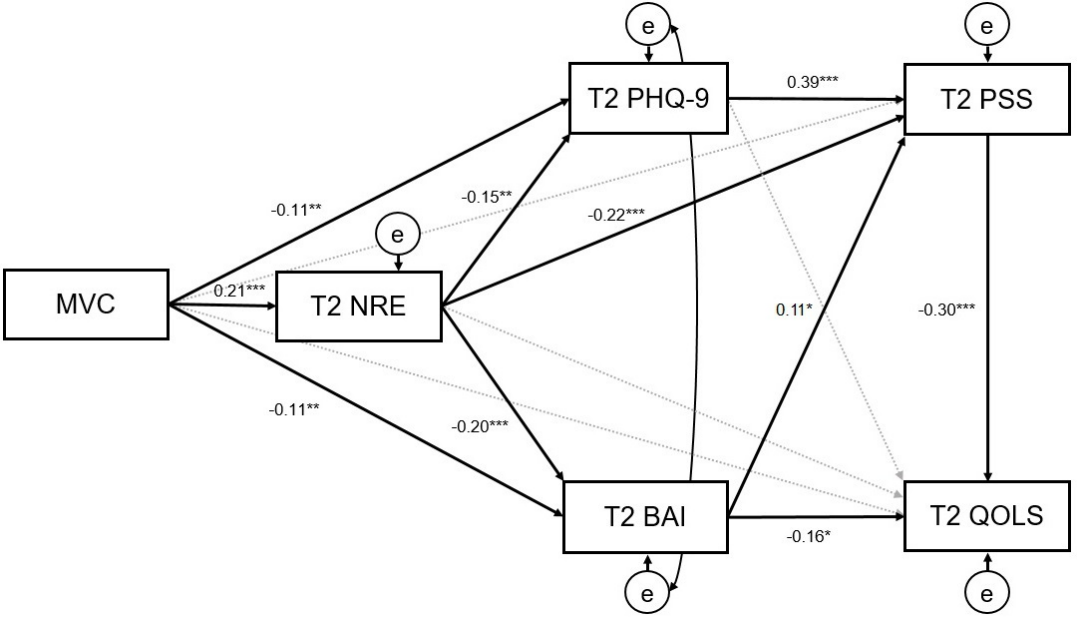
Mediation model indicating direct and indirect effects of MVC (compared to waitlist control) on study outcomes (β, *P<.05, **P<.01, ***P<.001; n= 306). BAI: Beck Anxiety Inventory; MVC Mindfulness Virtual Community; NRE: nonreactivity; PHQ-9: Patient Health Questionnaire-9; PSS: Perceived Stress Scale; QOLS: Quality of Life Scale; T1: baseline; T2: 8 weeks post intervention.

**Figure 2.**
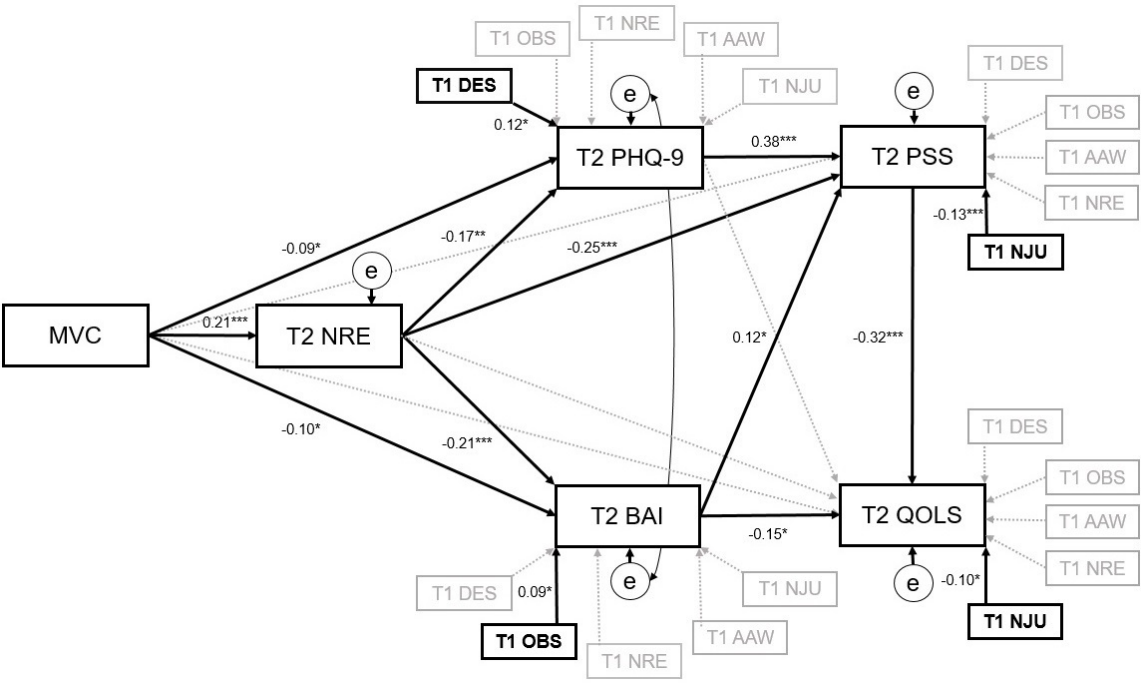
Mediation model (extended) indicating direct and indirect effects of Mindfulness Virtual Community (compared to waitlist control) on study outcomes, adjusted for preintervention Five Factor Mindfulness Questionnaire-Short Form (FFMQ-SF; β, *P<.05, **P<.01, ***P<.001; n= 306). AAW: acting with awareness; DES: describing; MVC: Mindfulness Virtual Community; NJU: nonjudgment; NRE: nonreactivity; OBS: observing; PHQ-9: Patient Health Questionnaire-9; PSS: Perceived Stress Scale; QOLS: Quality of Life Scale; T1: baseline; T2: 8 weeks post intervention.

Specification of the proposed model was guided by the intervention’s M-CBT theoretical perspective and evaluation of direct (ie, nonmediated) intervention effects on study outcomes. Subsequently, a conceptual model was specified (see Figure 1) to assess the role of NRE (as the sole FFMQ-SF facet with statistically significant direct effects) as a mediator of intervention effects on depression and anxiety (symptom-driven outcomes), and perceived stress and quality of life (as functional outcomes). For perceived stress, the model additionally explored depression and anxiety as mediators. For quality of life, the model included depression, anxiety, and perceived stress as mediators. The extended model (see Figure 2) included additional adjustments for other mindfulness facets at preintervention, to take into account possible individual differences and associations between FFMQ-SF facets and study outcomes. Both models specified covariances among exogenous variables (ie, preintervention levels of study outcomes). In both models, all proposed mediators and outcomes at postintervention (ie, 8 wk) were adjusted for their respective preintervention levels. Given elevated correlations between depression and anxiety at postintervention (*r*=.70 to *r*=.75 across imputed datasets), the covariance between their residual errors post intervention was additionally specified. Similarly, both depression and anxiety at postintervention were adjusted for each other’s baseline levels. 

### Model Fit

Model fit was evaluated according to multiple indices, including the comparative fit index (CFI), the root-mean-square error of approximation (RMSEA), and the standardized root-mean-square residual (SRMR). Models were deemed good or acceptable fit with a CFI≥.95, an RMSEA<.08, an SRMR<.08, and in line with standard recommendations [60].

### Ethical Considerations

This research was approved by the Human Participants Research Committee at York University (Certificate: 2023 - 012).

## Results

### Participants

Study participants were 306 undergraduate university students who were enrolled in the intervention program in 2017 (n=148) and 2018 (n=158) cohorts with complete psychometric evaluations at preintervention. Altogether, n=29 participants (9.5%) across both cohorts dropped out without completing postintervention assessments, with 18 from the 2017 and 11 from the 2018 cohorts. Although reasons for attrition were not indicated in original publications [47,48], attrition rate did not differ by cohort year (*P*=.12), and there were no statistically significant differences in demographics or psychological characteristics among the participants who dropped out and those who completed the 8-week MVC intervention.

As shown in Table 1, no statistically significant differences in demographic or psychological characteristics were detected at preintervention between MVC and WLC groups. These included mean age (*P*=.59), gender (*P*=.10), country of birth (*P*=.25), first language (*P*=.47), relationship status (*P*=.06), ethnic background (*P*=.90), PHQ-9 (*P*=.38), BAI (*P*=.80), PSS (*P*=.50), QOLS (*P*=.35), and FFMQ-SF facets (*P*=.17 to *P*=.71). In addition, when comparing preintervention demographic and psychological characteristics between participants from the years 2017 and 2018, no statistically significant differences were seen, except for higher proportions of single (no relationship) participants in the 2018 WLC group and married participants in the 2017 WLC group (*P=.*001).

**Table 1.** Demographic and psychological characteristics of study participants at preintervention.

Demographic characteristics	MVC^a^ (n=152)	WLC^b^ (n=154)	*P* value
Age, mean years (SD)	23.21 (7.53)	23.73 (9.23)	.59
Gender, n (%)			.10
Male	40 (26.3)	25 (16.2)	
Female	111 (73)	128 (83.1)	
Other	1 (0.7)	1 (0.6)	
Country of birth, n (%)			.25
Canada	80 (52.6)	71 (46.1)	
Other	72 (47.4)	83 (53.9)	
Years in Canada, mean (SD)^c^	9.8 (9.1)	9.0 (7.1)	.56
First language, n (%)			.47
English	95 (62.5)	90 (58.4)	
Other	57 (37.5)	64 (41.6)	
Relationship status, n (%)^d^			.06
Single	81 (53.3)	96 (62.3)	
Single (in relationship)	56 (36.8)	36 (23.4)	
Married or common law relationship	13 (8.6)	17 (11)	
Other	2 (1.3)	5 (3.2)	
Ethnic background, n (%)			.90
White	29 (19.1)	27 (17.5)	
Black	21 (13.8)	18 (11.7)	
South Asian	37 (24.3)	42 (27.3)	
Other	49 (32.2)	47 (30.5)	
Multiple ethnicities	16 (10.5)	20 (13)	
Self-rated health, n (%)			.27
Poor or fair	26 (17.1)	36 (23.4)	
Good	54 (35.5)	57 (37)	
Very good or excellent	72 (47.4)	61 (39.6)	
Access to private mental health, n (%)			.42
Yes	59 (38.8)	53 (34.4)	
No	93 (61.2)	101 (65.6)	
Weekly hours, mean hours (SD)			
Paid work	9.32 (9.77)	9.13 (11.44)	.87
Unpaid work (incl. volunteer work)	2.71 (4.76)	3.45 (5.69)	.22
Vigorous physical activities^e^	2.64 (5.51)	2.22 (5.4)	.50
Psychological Characteristics^d^			
PHQ-9, mean (SD)^f^	8.71 (6.4)	9.36 (6.49)	.38
PHQ-9 score ranges, n (%)			.63
0‐9	90 (59.2)	87 (56.5)	
≥ 10	62 (40.8)	67 (43.5)	
BAI, mean (SD)^g^	15.18 (12.1)	15.51 (11.39)	.80
BAI range, n (%)			.76
0‐21 (low)	111 (73)	110 (71.4)	
≥ 22 (moderate-high)	41 (27)	44 (28.6)	
PSS score, mean (SD)^h^	19.97 (7.78)	20.56 (7.55)	.50
PSS score ranges, n (%)			.76
0‐13 (low)	28 (18.4)	27 (17.5)	
14‐26 (moderate)	91 (59.9)	88 (57.1)	
27‐40 (high)	33 (21.7)	39 (25.3)	
QOLS, mean (SD)^i^	81.15 (15.95)	79.51 (15.22)	.35
FFMQ-SF, mean (SD)^j^			
DES^k^	16.10 (4.16)	16.58 (4.31)	.31
OBS^l^	13.59 (3.43)	13.88 (3.27)	.44
AAW^m^	16.44 (4.31)	16.24 (4.72)	.71
NJU^n^	14.12 (3.9)	13.95 (4.37)	.70
NRE^o^	13.85 (3.97)	14.44 (3.68)	.17

a MVC: Mindfulness Virtual Community.

b WLC: waitlist control.

c Based on 155 participants born outside of Canada.

d The other category includes engaged, divorced, separated, or widowed.

e Based on 303 participants.

f PHQ-9: Patient Health Questionnaire-9.

g BAI: Beck Anxiety Inventory.

h PSS: Perceived Stress Scale.

i QOLS: Quality of Life Scale.

j FFMQ-SF: Five Facet Mindfulness Questionnaire-Short Form.

k DES: describing.

l OBS: observing.

m AAW: acting with awareness.

n NJU: nonjudgment.

o NRE: nonreactivity.

### Between-Group Differences in Study Outcomes

Table 2 presents means (SD) for MVC and WLC groups at pre (ie, baseline) and at 8 weeks post intervention. The effect of study group (ie, MVC vs WLC) on outcomes (ie, direct nonmediated effects) was indicated by statistically significant between-group differences in study outcomes post intervention, adjusted for baseline levels of the respective outcomes. Results indicated statistically significant differences between MVC and WLC groups post intervention on the PHQ-9 (*b*=−1.72; *P*=.002), BAI (*b*=−3.40; *P*=.001), PSS (*b*=−2.44; *P*<.001), QOLS (*b*=4.31; *P*=.005), and the NRE facet of the FFMQ-SF (*b*=1.63; *P*<.001), all favoring the MVC.

**Table 2.** Postintervention differences (8 weeks) in study outcomes between Mindfulness Virtual Community (MVC) and Waitlist Control (WLC) groups.

Outcomes and time			MVC^a^ (n=152)	WLC^b^ (n=154)	B (95% CI)^c^	β	*P* value
PHQ-9^d^					−1.72 (−2.78 to −0.64)	−0.13	.002
	Preintervention		8.71 (6.40)	9.36 (6.49)			
	Postintervention		7.81 (6.14)	9.98 (6.82)			
BAI^e^					−3.40 (−5.42 to −1.37)	−0.15	.001
	Preintervention		15.18 (12.10)	15.51 (11.39)			
	Postintervention		11.84 (10.69)	15.45 (11.86)			
PSS^f^					−2.44 (−3.78 to −1.10)	−0.16	<.001
	Preintervention		19.97 (7.78)	20.56 (7.55)			
	Postintervention		18.30 (7.03)	21.11 (8.00)			
QOLS^g^					4.31 (1.29 to 7.32)	0.13	.005
	Preintervention		81.15 (15.95)	79.51 (15.22)			
	Postintervention		80.80 (15.33)	75.42 (17.15)			
FFMQ-SF^h^							
	DES^i^				0.37 (−0.35 to 1.09)	0.05	.32
		Preintervention	16.10 (4.16)	16.58 (4.31)			
		Postintervention	16.40 (3.67)	16.33 (4.34)			
	OBS^j^				0.60 (−0.04 to 1.25)	0.09	.07
		Preintervention	13.59 (3.43)	13.88 (3.27)			
		Postintervention	13.74 (3.66)	13.32 (3.34)			
	AAW^k^				0.25 (−0.51 to 1.02)	0.03	.52
		Preintervention	16.44 (4.31)	16.24 (4.72)			
		Postintervention	15.80 (3.75)	15.46 (3.93)			
	NJU^l^				−0.07 (−0.86 to 0.71)	−0.009	.85
		Preintervention	14.12 (3.90)	13.95 (4.37)			
		Postintervention	14.12 (4.07)	14.10 (4.26)			
	NRE^m^				1.63 (0.89 to 2.37)	0.22	<.001
		Preintervention	13.85 (3.97)	14.44 (3.68)			
		Postintervention	15.71 (3.57)	14.38 (3.89)			

a MVC: Mindfulness Virtual Community.

b WLC: waitlist Control.

c Between-group differences at postintervention, adjusted for each outcome’s baseline levels. b and β (unstandardized and standardized estimates respectively) indicate the direct (ie, nonmediated) effect of the MVC intervention on study outcomes.

d PHQ-9: Patient Health Questionnaire-9.

e BAI: Beck Anxiety Inventory.

f PSS: Perceived Stress Scale.

g QOLS: Quality of Life Scale.

h FFMQ-SF: Five Facet Mindfulness Questionnaire-Short Form.

i DES: Describing.

j OBS: Observing.

k AAW: acting with awarenesss.

l NJU: Nonjudgment.

m NRE: Nonreactivity.

### Mediation Models

Following evaluation of post-intervention differences between MVC and WLC groups (ie, adjusted direct effects), NRE, as the sole FFMQ-SF facet impacted by the MVC intervention, was included in the mediation model. In accordance with the hypothesized model (see Figure 1 and Table 3), results supported NRE as a mediator of the effects of MVC intervention on depression (*b*=−.41; *P*=.03), anxiety (*b*=−.93; *P*=.003), and perceived stress (*b*=−.70; *P*=.002), but not quality of life (*b*=.34; *P*=.32).

**Table 3.** Direct and indirect paths per study outcome in the main mediation model.

Outcomes and paths		B (95% CI)	β	*P* value
Depression				
	MVC^a^>NRE	1.63 (0.86‐2.40)	0.21	<.001
	NRE^b^>PHQ-9	−0.25 (−0.42 to −0.08)	−0.15	.004
	MVC>NRE>PHQ-9^c^	−0.41 (−0.79 to −0.11)	—^d^	.03
Anxiety				
	NRE>BAI^e^	−0.57 (−0.85 to −0.30)	−0.20	<.001
	MVC>NRE>BAI	−0.93 (−1.67 to −0.36)	—	.003
Perceived stress				
	NRE>PSS^f^	−0.43 (−0.59 to −0.27)	−0.22	<.001
	MVC>NRE>PSS	−0.70 (−1.18 to −0.31)	—	.002
	MVC>PHQ-9	−1.44 (−2.54 to −0.34)	−0.11	.01
	PHQ-9>PSS	0.45 (0.31‐0.58)	0.39	<.001
	MVC>PHQ-9>PSS	−0.64 (−1.24 to −0.14)	—	.02
	MVC>NRE>PHQ-9>PSS	−0.18 (−0.37 to −0.05)	—	.03
	MVC>BAI	−2.48 (−4.44 to −0.52)	−0.11	.01
	BAI>PSS	0.07 (0.003‐0.14)	0.11	.04
	MVC>BAI>PSS	−0.18 (−0.45 to 0.001)	—	.11
	MVC>NRE>BAI>PSS	−0.07 (−0.17 to −0.003)	—	.09
Quality of life				
	NRE>QOLS	0.21 (−0.18 to 0.59)	0.05	.29
	MVC>NRE>QOLS^g^	0.34 (−0.29 to 1.04)	—	.32
	PHQ-9>QOLS	−0.10 (−0.48 to 0.29)	−0.04	.62
	MVC>PHQ-9>QOLS	0.14 (−0.47 to 0.76)	—	.61
	MVC>NRE>PHQ-9>QOLS	0.04 (−0.12 to 0.24)	—	.68
	BAI>QOLS	−0.23 (−0.44 to −0.02)	−0.16	.03
	MVC>BAI>QOLS	0.56 (0.002‐1.50)	—	.05
	MVC>NRE>BAI>QOLS	0.21 (0.01‐0.53)	—	.03
	MVC>PSS	−0.83 (−1.96 to 0.31)	-0.06	.15
	PSS>QOLS	−0.65 (−0.91 to −0.38)	-0.30	<.001
	MVC>PSS>QOLS	0.54 (−0.20 to 1.35)	—	.21
	MVC>NRE>PSS>QOLS	0.45 (0.17‐0.85)	—	.02
	MVC>PHQ-9>PSS>QOLS	0.41 (0.08‐0.93)	—	.05
	MVC>BAI>PSS>QOLS	0.12 (−0.001 to 0.32)	—	.16
	MVC>NRE>PHQ-9>PSS>QOLS	0.12 (0.03‐0.27)	—	.08
	MVC>NRE>BAI>PSS>QOLS	0.04 (0.002‐0.12)	—	.14
	MVC>QOLS	1.54 (−1.00 to 4.07)	0.05	.23

a MVC: Mindfulness Virtual Community.

b NRE: Nonreactivity.

c PHQ-9: Patient Health Questionnaire-9.

d Not available.

e BAI: Beck Anxiety Inventory.

f PSS: Perceived Stress Scale.

g QOLS: Quality of Life Scale.

For perceived stress, results additionally supported the mediating role of depression (*b*=−.64; *P*=.02), but not anxiety (*b*=−.18; *P*=.11). For quality of life, results indicated mediation through anxiety (*b*=.57*; P*=.05), but not depression (*b*=.14; *P*=.61). The effects of MVC intervention on PSS were further mediated by a sequential mediation path through NRE and PHQ (*b*=−.18; *P*=.03). Similarly, quality of life was mediated by sequential mediation paths through NRE and BAI (*b*=.21; *P*=.03), and NRE and PSS (*b*=.45; *P*=.02; see Table 3).

Interestingly, in the presence of mediated (ie, indirect) effects, the direct paths from the MVC intervention to depression (*b*=−1.44; *P=*.01) and anxiety (*b*=−2.48; *P*=.01) reduced in magnitude when compared to direct effect models (see Table 2). Similarly, direct paths from the MVC intervention to perceived stress (*b*=−0.83; *P*=.15) and quality of life (*b*=1.54; *P*=.23) were no longer statistically significant. Model fit indices indicated acceptable fitness (CFI=.988; RMSEA=.052; SRMR=.05). The model accounted for 52.1% of variability in depression, 49.3% of variability in anxiety, 64.5% of variability in perceived stress, and 57.5% of variability in quality of life.

In the extended model (see Figure 2 and Table 4), all outcomes were further adjusted for preintervention levels of mindfulness (ie, FFMQ-SF) facets. Given that mindfulness is both regarded as an individual disposition (ie, trait) and a cultivated capacity, an extension of the model additionally accounted for individual differences in dispositional mindfulness. Overall, results from the extended model (see Figure 2 and Table 4) were similar to the main model (see Figure 1 and Table 3). However, preintervention FFMQ-SF facets showed different patterns of association with study outcomes. Specifically, among the 5 mindfulness facets at preintervention, statistically significant relationships were observed between DES and depression (*b*=.19; *P=*.02), OBS and anxiety (*b*=.30; *P=*.05), NJU and perceived stress (*b*=−.23; *P*=.001), NJU and quality of life (*b*=−.37; *P*=.03). Model fit indices for the extended model indicated good fitness (CFI=.992; RMSEA=.043; SRMR=.036).

**Table 4.** Direct and indirect paths per study outcome in the extended mediation model.

Outcomes and paths		B (95% CI)	β	*P* value
Depression				
	MVC^a^>NRE^b^	1.63 (0.87‐2.39)	0.21	<.001
	NRE>PHQ-9^c^	−0.30 (−0.49 to −0.13)	−0.17	.004
	MVC>NRE>PHQ-9	−0.48 (−0.91 to −0.13)	—^d^	.02
Anxiety				
	NRE>BAI^e^	−0.63 (−0.93 to −0.33)	−0.21	<.001
	MVC>NRE>BAI	−1.02 (−1.80 to −0.41)	—	.002
Perceived stress				
	NRE>PSS^f^	−0.48 (−0.64 to −0.32)	−0.25	<.001
	MVC>NRE>PSS	−0.78 (−1.30 to −0.36)	—	.001
	MVC>PHQ-9	−1.22 (−2.37 to −0.06)	−0.09	.04
	PHQ-9>PSS	0.44 (0.30‐0.57)	0.38	<.001
	MVC>PHQ-9>PSS	−0.53 (−1.13 to −0.02)	—	.04
	MVC>NRE>PHQ-9>PSS	−0.21 (−0.43 to −0.05)	—	.03
	MVC>BAI	−2.28 (−4.23 to −0.32)	−0.10	.02
	BAI>PSS	0.08 (0.008‐0.15)	0.12	.03
	MVC>BAI>PSS	−0.18 (−0.44 to −0.001)	—	.13
	MVC>NRE>BAI>PSS	−0.08 (−0.19 to −0.008)	—	.08
Quality of life				
	NRE>QOLS^g^	0.34 (−0.09 to 0.76)	0.08	.12
	MVC>NRE>QOLS	0.55 (−0.13 to 1.40)	—	.17
	PHQ-9>QOLS	−0.12 (−0.49 to 0.25)	−0.05	.52
	MVC>PHQ-9>QOLS	0.15 (−0.35 to 0.72)	—	.53
	MVC>NRE>PHQ-9>QOLS	0.06 (−0.12 to 0.29)	—	.60
	BAI>QOLS	−0.21 (−0.41 to −0.007)	−0.15	.04
	MVC>BAI>QOLS	0.48 (−0.01 to 1.37)	—	.06
	MVC>NRE>BAI>QOLS	0.22 (0.005‐0.53)	—	.03
	MVC>PSS	−0.73 (−1.84 to 0.38)	−0.05	.20
	PSS>QOLS	−0.70 (−0.97 to −0.43)	−0.32	<.001
	MVC>PSS>QOLS	0.51 (−0.27 to 1.36)	—	.24
	MVC>NRE>PSS>QOLS	0.55 (0.23‐0.99)	—	.007
	MVC>PHQ-9>PSS>QOLS	0.37 (0.01‐0.89)	—	.08
	MVC>BAI>PSS>QOLS	0.13 (0.00‐0.34)	—	.18
	MVC>NRE>PHQ-9>PSS>QOLS	0.15 (0.03‐0.33)	—	.06
	MVC>NRE>BAI>PSS>QOLS	0.06 (0.005‐0.14)	—	.12
	MVC>QOLS	1.06 (−1.52 to 3.65)	0.03	.42

a MVC: Mindfulness Virtual Community

b NRE: nonreactivity.

c PHQ-9: Patient Health Questionnaire-9.

d Not available.

e BAI: Beck Anxiety Inventory.

f PSS: Perceived Stress Scale.

g QOLS: Quality of Life Scale.

## Discussion

### Principal Findings

In this study we evaluated a mediation model for the MVC intervention, aimed at clarifying its therapeutic mechanisms, based on data from 2 RCTs involving undergraduates at a Canadian university. The mediation model (see Figure 2 and Table 3) supported the role of the NRE facet of FFMQ-SF as a mediator of the MVC intervention effects on depression, anxiety, perceived stress, and quality of life outcomes. Our results further supported the role of depression as a mediator for the MVC intervention effects on perceived stress, alongside other sequential mediation paths for perceived stress and quality of life. Changes in NRE reflect an important intervention effect, indicating participants’ increased capacities to discontinue automatic depressogenic-anxiogenic reactions to events. This is exemplified in the mindfulness of breath technique emphasized, where participants were instructed to direct awareness to breathing sensations and return attention to them after thoughts and emotions arise, creating a “gap” between stimuli exposure and automatic reaction. This gap introduces a context for altering automatic responding, resulting in responses that reflected increased awareness and decreased avoidance. Therefore, phenomena that may have previously been viewed automatically (in a negative frame) could now be re-perceived in open-ended, neutral, and potentially positive ways.

According to Burzler and Tran’s [61] proposed model of mindfulness development with respect to the five facets model, mindfulness cultivation involves progressive developments in OBS, DES*,* and AAW facets, indicating enhanced attentional deployment, which then stimulates further development of the NJU and NRE facets. Burzler and Tran’s [61] model emphasizes development in the attentional elements of the construct (ie, OBS, DES, and AAW) before development of the attitudinal elements (ie, NJU and NRE). While our findings were similar to their proposed model in supporting NRE as the final outcome of mindfulness training, a comprehensive evaluation of this model requires determination of change trajectories of individual facets over the course of intervention with multiple measurements in closer succession (eg daily or weekly evaluation). In contrast to this model, there is also evidence that in mindfulness development, different attentional and attitudinal facets may interact to produce healthy outcomes. For example, the OBS facet has been shown to be associated with better sleep health at higher NRE levels but not at lower NRE levels [62]. Our findings emphasize the NRE facet as the key mediator of the MVC intervention. This is aligned with previous evaluations that identified NRE as a mediator for improvements in cognitive flexibility in mindfulness-based stress reduction training [63], and reductions in health anxiety in cognitive-behavioral and acceptance and commitment therapies [64,65]. Further support for the importance of NRE to well-being and distress emerged in a study on the relative importance of FFMQ facets as dispositions in a general population sample [66]. Specifically, in that study, NRE accounted for the highest proportion of variance (25%) in well-being, and the second highest proportion of variance (7%) in distress after AAW (20%) [66].

In contrast to signifying passivity, we suggest NRE, as conceptualized in the FFMQ-SF, reflects an attitude of nonavoidance with equanimity, defined as even-minded responsiveness to mental phenomena, unswayed by associated emotional valences [67]. Indeed, NRE has shown the highest magnitude of correlation with the even-mindedness subscale of the EQUA-S equanimity scale (.54; *P*<.001), among other FFMQ facets (AAW: .22; *P*<.001, NJU: .30; *P*<.001) [68]. NRE has further shown a moderate negative correlation with experiential avoidance (−.39; *P*<.001), alongside other FFMQ facets (DES: −.23, *P*<.001; AAW: −.30, *P*<.001; NJU: −.49, *P*<.001) [40]. In the same study, both NRE (.53, *P*<.001) and NJU (.48, *P*<.001) demonstrated the highest correlations with self-compassion [40].

### Strengths and Limitations

There are advantages and limitations to this investigation. First, our study used a large sample size based on data aggregated from two RCTs with identical recruitment and administration procedures. In addition, among previous evaluations of specific mindfulness facets as mediating mechanisms of MBIs [21-23,44], our research provides key evidence on the role of NRE as a mediator of MBI with an M-CBT perspective. Second, we used a structural equation modeling approach to evaluate mediation, which, in comparison to regression-based methods, permits simultaneous evaluations of multiple inter-relationships of varying complexities (ie, parallel and sequential indirect paths). In this approach, as was the case for depression, anxiety, and perceived stress in this study, variables can serve as both mediators and outcomes within a hypothesized matrix of associations. However, as the MVC intervention evaluated outcomes at 2 time points, absence of temporal precedence of mediators in this study precludes a definite indication of causality [69]. Longitudinal evaluations with multiple time points are better suited to establish the temporal precedence of change in mediators before change in outcomes.

The mediation models in this study demonstrated good fit, in accordance with fit indices guidelines, and accounted for between 43.1% and 62.9% of variation in study outcomes, leaving room for the evaluation of other putative mechanisms. Regarding other mechanisms of interest, considering that mindfulness meditation practice and MBIs broadly emphasize mindfulness as a metacognitive skill and awareness [11,67,70], future research can benefit from exploring the mediating role of maladaptive metacognitive tendencies. The Metacognitions Questionnaire-30 (MCQ-30) provides an interesting target for future research in this regard [71].

While evaluations of metacognitive mediators are scarce at present, two studies can provide preliminary insights into associations between mindfulness and metacognitive beliefs. First, Love et al [72] evaluated associations between mindfulness facets, metacognitive tendencies, and experience of flow among athletes, revealing different patterns of predictive relationships between MCQ-30 subscales and FFMQ-SF facets. For NRE specifically, these included statistically significant inverse relationships with MCQ-30 subscales of positive (*b*=−3.27, *P*=.03) and negative (*b*=−7.67, *P*<.001) beliefs about worry, and positive relationships with cognitive self-consciousness (*b*=.23, *P*<.001). Though preliminary, these associations show an interesting pattern, in which NRE exhibited a positive relationship with awareness of cognitions (ie, cognitive self-consciousness), while also showing inverse relationships with either positive or negative metacognitive beliefs. These findings highlight a quality of greater cognitive awareness, influenced by fewer positive or negative worry-based metacognitive beliefs. More recently also, Lefebvre et al [73] evaluated the role of meta-cognitive beliefs, as evaluated by MCQ-30, and mindfulness among depressed couples and found the MCQ-30 mediated associations between depression severity, affective expression, and mindfulness facets.

This research aggregated data from the 2017 and 2018 cohorts of the MVC research study. Although these research studies recruited study samples from the same student population and followed similar administrative procedures, we conducted sensitivity analysis to explore whether study models were overly influenced by cohort year. Overall, results of the stratified analysis were similar to the overall analysis in terms of the magnitude and direction of effects, despite changes to statistical significance levels. Considering that structural equation modeling is a large-sample method [74,75], we consider higher *P* values in separate analyses of the 2017 and 2018 cohorts to be a reflection of smaller sample sizes. In calculations of required sample size for an expected magnitude of effect, smaller magnitude effects often require larger sample sizes [76]. Since our observed effects (ie, β) were small to medium ranged, we consider the loss of sample size when study data were limited to 2017 or 2018 cohorts to be the reason for the increase in *P* values.

A further point of concern relates to possible between-group differences in associations between mediators and outcomes in the model. Specifically, while paths between study group (ie, MVC vs WLC) and mediators indicate a between-group difference (*a* paths in mediation terminology), paths between mediators and outcomes (*b* paths in mediation terminology) are not differentiated by group. This is because we did not assume the relationships between mediators and outcomes to differ between study groups and follow the same hypothesized direction (eg, increased depression to be associated with increased perceived stress, regardless of group assignment). We further tested this assumption by inclusion of interaction terms for these paths, assessing whether these relationships are moderated by group assignment. No statistically significant interactions were detected, further supporting the main hypothesized model.

### Conclusion

The primary clinically relevant insight from these findings pertains to the shift from automatic, and often avoidant, responding to distressing thoughts and emotions (before mindfulness meditation training) to responding with mindful nonreacting, as conceptualized by the NRE facet of the FFMQ-SF. While avoidance involves a shift in attention away from distressing thoughts, emotions, and events without intentions to return to re-perceive the immediate responses, mindfulness training facilitates a nonreactive and nonavoidant stance toward thoughts, emotions, and events as they arise. Therefore, a new relationship with experience is cultivated, characterized by greater openness to mental phenomena “as they are,” rather than anxious-depressive responding.

## Abbreviations

AAW: acting with awareness
BAI: Beck Anxiety Inventory
CFI: comparative fit index
DES: describing
FFMQ: Five Facet Mindfulness Questionnaire
FFMQ-SF: Five Factor Mindfulness Questionnaire-Short Form
M-CBT: mindfulness and cognitive-behavioral therapy
MBI: mindfulness-based intervention
MCQ-30: Metacognitions Questionnaire-30
MICE: Multivariate Imputation by Chained Equations
MVC: Mindfulness Virtual Community
NJU: nonjudgment
NRE: nonreactivity
OBS: observing
PHQ-9: Patient Health Questionnaire-9
PSS: Perceived Stress Scale
QOLS: Quality of Life Scale
RCT: randomized controlled trial
RMSEA: root-mean-square error of approximation
SRMR: standardized root-mean-square residual
WLC: waitlist control
